# Precision medicine in liver transplantation for hepatocellular carcinoma: applications and prospects of third-generation sequencing technology

**DOI:** 10.1007/s00432-025-06299-3

**Published:** 2025-09-13

**Authors:** Ye Tian, Xiaojuan Wang, Qian Lu

**Affiliations:** 1 Hepatopancreatobiliary Center, Beijing Tsinghua Changgung Hospital, Key Laboratory of Digital Intelligence Hepatology (Ministry of Education), School of Clinical Medicine, Tsinghua Medicine, Tsinghua University, 102218 Beijing, China; 2 Institute for Organ Transplant and Bionic Medicine, Beijing Key Laboratory of Liver Transplantation and Bionic Manufacturing, Tsinghua University, 102218 Beijing, China

**Keywords:** Precision medicine, Third-generation sequencing (TGS), Hepatocellular carcinoma (HCC), Liver transplantation (LT)

## Abstract

Hepatocellular carcinoma (HCC) is one of the leading causes of cancer-related deaths worldwide. Liver transplantation (LT) remains a vital treatment for HCC, yet it still faces numerous challenges in patient selection, recurrence monitoring, and personalized therapy. Third-generation sequencing (TGS), with its advantages of long read length, high throughput, direct detection of epigenetic modifications, real-time analysis and high accuracy, offers promise for advancing precision medicine in LT. While previous reviews have focused on TGS technical features, this review uniquely synthesizes its role in addressing specific clinical challenges in LT-HCC management and critically assesses its translational pathway. Specifically, it systematically examines TGS applications in candidate screening, recurrence monitoring, and personalized therapy for LT-HCC, analyzes its prospects for clinical translation, and aims to provide new insights for precise HCC treatment. We also discuss barriers to implementation and suggest solutions for integrating TGS into clinical workflows. This review provides a roadmap for leveraging TGS to revolutionize precision medicine in transplant hepatology.

## Introduction

Hepatocellular carcinoma (HCC), the leading type of primary liver cancer, significantly contributes to increasing global cancer mortality and requires innovative management strategies (Llovet et al. 2021). While liver transplantation (LT) offers a potential cure for early-stage HCC patients who meet Milan or University of California, San Francisco (UCSF) criteria (Li et al. 2024), critical challenges remain in clinical practice: patients exceeding traditional size or number limits may still benefit from LT but lack validated molecular selection tools (Zhou et al. 2025); waitlist dropout due to tumor progression affects 20–25% of candidates, highlighting the need for dynamic monitoring approaches (Mehta et al. 2021); post-LT recurrence (in 15–20% of recipients) remains the main cause of death, yet conventional biomarkers (AFP, PIVKA-II) show limited sensitivity for early detection (Zhang et al. 2017; Özdemir and Baskiran 2020); and histopathology assessments cannot fully represent tumor heterogeneity, limiting personalized neoadjuvant or adjuvant therapeutic strategies (Zhang et al. 2017). These challenges emphasize the urgent need for more accurate prognostic tools and detection methods to improve HCC management for transplant candidates and recipients.

Third-generation sequencing (TGS) technologies—including single-molecule real-time (SMRT) and Nanopore sequencing (Liu et al. 2024a)—are uniquely equipped to address these clinical gaps through three key advantages over traditional sequencing: (1) Long reads (> 10 kb) allow for thorough resolution of structural variations (SVs) and fusion genes essential for prognostication (Hu et al. 2022); (2) Direct detection of base modifications (e.g., 5-methylcytosine) enables non-invasive epigenetic profiling without bisulfite-induced DNA degradation (Pai et al. 2022; Sigurpalsdottir et al. 2024); and (3) Real-time data analysis from portable platforms (e.g., Oxford Nanopore) supports rapid liquid biopsy applications during waitlist monitoring (Athanasopoulou et al. 2021).

This review summarizes recent advances in TGS-driven precision medicine across three key clinical areas: (1) pre-LT decision-making using molecular subtyping beyond anatomical criteria; (2) post-LT management with ultra-sensitive circulating tumor DNA (ctDNA) analysis and immune monitoring to predict recurrence or rejection; and (3) translational pathways that address barriers to clinical adoption. By critically assessing current evidence and technical challenges, we offer a roadmap for integrating TGS into the LT-HCC workflow.

## The application of TGS in liver transplantation for liver cancer

### Overview of TGS technology

**Fig. 1:**
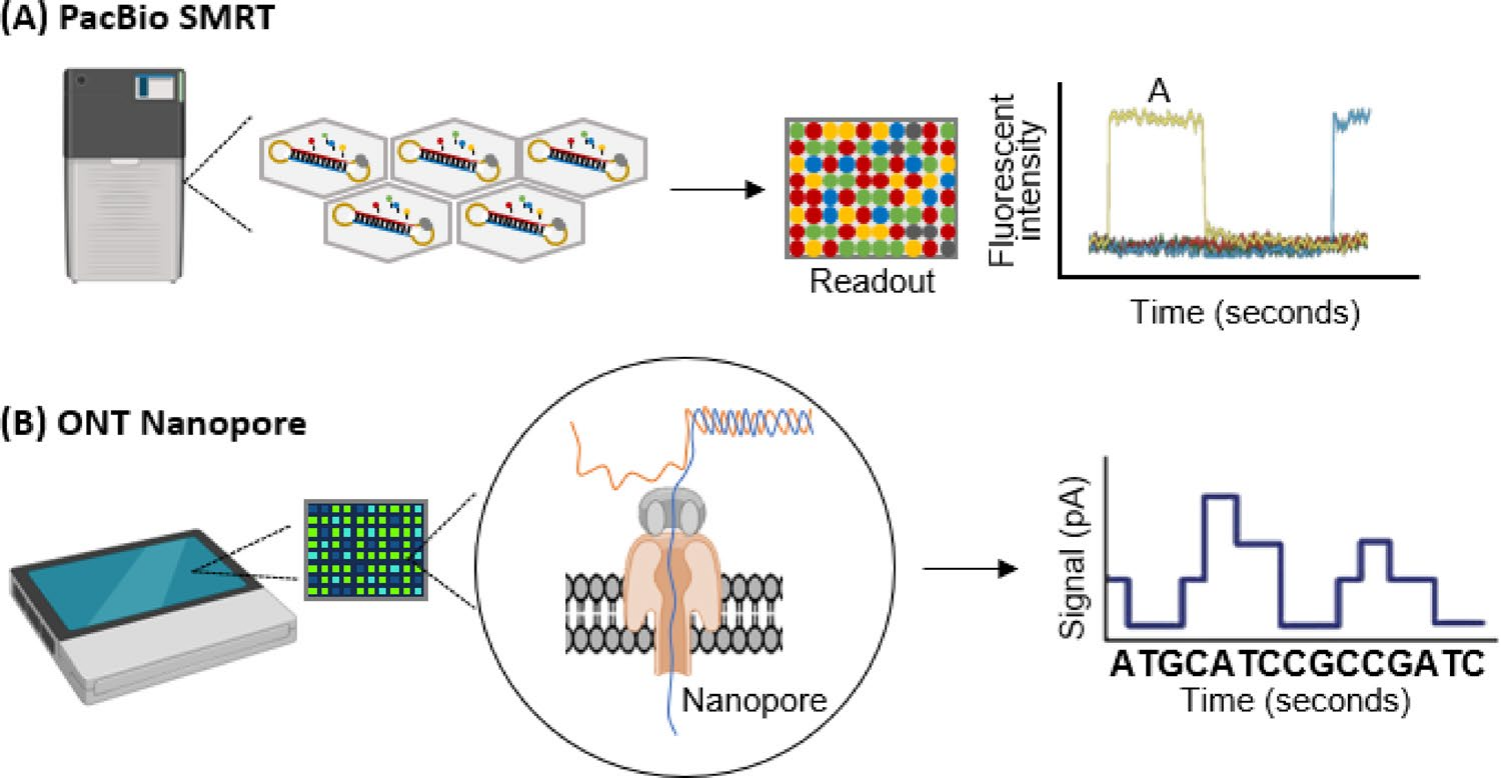
TGS technologies by SMRT (PacBio) and Nanopore (ONT). **A** SMRT (PacBio): Sequencing occurs by immobilizing single DNA polymerase molecules in ZMWs and optically detecting fluorescent signals emitted during nucleotide incorporation. **B** Nanopore (ONT): Sequencing is achieved by driving single-stranded DNA through a Nanopore and identifying characteristic disruptions in ionic current caused by individual nucleotides

SMRT by Pacific Biosciences (PacBio) and Nanopore sequencing by Oxford Nanopore Technologies (ONT) are the two leading TGS technologies (Fig. 1). The SMRT technology is based on fixed single DNA polymerase molecules in zero-mode waveguides (ZMWs), and directly reads the DNA template sequence by monitoring the optical signals released when the enzyme binds to fluorescently labeled nucleotides during the synthesis process (Ardui et al. 2018). The other mainstream technology, Nanopore sequencing, relies on allowing single DNA molecules to pass through Nanopores, and directly identifies the sequence by detecting the characteristic current disturbance changes caused by different nucleotides passing through (Rukes and Cao 2025). Both technologies avoid the biases introduced by traditional PCR amplification and can directly read ultra-long fragments of tens of thousands to several hundred thousand bases, significantly enhancing the assembly ability for complex genomes and effectively supporting structural variation analysis and resequencing applications. Compared with SMRT technology, which has higher repeatability and reproducibility, Nanopore sequencing, with its outstanding real-time and portability advantages, is particularly suitable for on-site rapid detection (Espinosa et al. 2023).

### TGS in pre-transplantation decision-making: molecular stratification beyond Milan criteria

#### Genomic structural variation burden: a novel prognostic marker

Genomic structural variations (SVs), generally defined as insertions (INSs), deletions (DELs), duplications (DUPs), inversions (INVs), and translocations (TRAs) (George et al. 2015), are considered significant biomarkers in HCC (Lyu et al. 2025). Duan and Chen et al. demonstrated that the HCC genome is characterized by a high frequency of SVs, which are associated with the invasive phenotype of the tumor and patient prognosis (Duan et al. 2019; Chen et al. 2024). Fujimoto et al. employed Nanopore long-read sequencing to analyze the whole genomes of 11 HCC and matched normal samples, developing specialized computational pipelines for comprehensive SV detection. This long-read approach revealed that germline SVs predominantly originate from transposon/Alu insertions and SINE-mediated non-allelic homologous recombination (NAHR) deletions. Importantly, Nanopore sequencing detected 420 SV candidates not identified by traditional methods, indicating that Nanopore sequencing outperforms previous short-read sequencing in detecting SVs (Fujimoto et al. 2021). Clinically, SV burden can be used to independently predict the biological behavior of patients beyond the Milan criteria, offering new possibilities for personalized modified criteria and providing a basis for more precise treatment plans. Additionally, it demonstrates the potential of TGS in molecular stratification.

#### Gene fusion and driving events: identification of latent high-risk subtypes

In the molecular stratification of liver cancer, identifying fusion genes is of vital importance. The TGS, with its long-read capabilities, can accurately detect fusion events that traditional methods often miss, opening new possibilities for identifying high-risk subtypes. Using Nanopore sequencing, Kiyose et al. analyzed 42 HCC/non-tumor liver pairs and discovered novel fusion transcripts, including LINE1-MET and HBV-transposable element chimeras, which were undetectable by short-read technologies. Functional validation confirmed that these Nanopore-detected fusions promote tumor growth (Kiyose et al. 2022). This finding underscores the importance of identifying fusion genes in liver cancer, especially for patients initially considered non-transplantable. The therapeutic relevance for these high-risk subtypes is that new adjuvant therapies with MET inhibitors (such as Savolitinib) can significantly shrink tumors (Wang et al. 2024a), potentially enabling patients who were previously ineligible for transplant to become candidates. This advancement not only offers new avenues for personalized treatment of liver cancer patients but also highlights the potential of precision medicine in tumor management.

#### Epigenetic profiling: methylation profiles predict microvascular invasion

Epigenetics also highlights its unique role in liver cancer prognosis, especially in early recurrence. Han et al. identified hypermethylated CpG sites in HCC, with cg02746869 standing out as a key regulatory site for VIM-AS1 lncRNA, where methylation status correlated with microvascular invasion (MVI) and early recurrence risk (Han et al. 2024). Second-generation sequencing depends on indirect methods like bisulfite treatment or antibody enrichment to detect DNA methylation, while TGS can directly identify base modifications (such as 5mC) through real-time signals of natural DNA templates, avoiding DNA damage and bias caused by bisulfite treatment (Flynn et al. 2022; Liu et al. 2025). Additionally, liquid biopsy technology allows non-invasive assessment of MVI status through plasma cfDNA methylation tags, eliminating potential sampling biases (Afflerbach et al. 2024). This technological progress not only improves the predictive power for MVI but also offers a new approach for early diagnosis of liver cancer, demonstrating epigenetics' potential in precision medicine for liver cancer.

### TGS in post-transplant management: recurrence warning and immune evaluation

#### ctDNA monitoring: recurrence prediction

The recurrence of liver cancer after transplantation is a key factor influencing long-term patient survival. Circulating tumor DNA (ctDNA), as an emerging biomarker, is becoming an important tool for detecting recurrence and enabling precise intervention (Abdelrahim et al. 2025; Loszko et al. 2025). The use of TGS has significantly enhanced the monitoring sensitivity of ctDNA. Marcozzi et al. developed a nanopore-based technology that increased sensitivity of ctDNA detection by approximately 60-fold, achieving an unprecedented detection limit of 0.02% for TP53 mutations and allowing for precise real-time monitoring of treatment response (Marcozzi et al. 2021). This approach highlights the unique ability of Nanopore technology for ultra-sensitive liquid biopsy applications in cancer monitoring. For liver transplant patients, this means that signals of tumor recurrence can be detected earlier, providing a critical window for clinical intervention.

Furthermore, the recurrence mechanism in liver cancer is closely linked to tumor genomic heterogeneity and copy number variations (CNVs). TGS technology, with its advantage of long-read length, offers a new tool for analyzing this complex biological process. Specifically, previous studies have used Nanopore sequencing technology, combined with shallow whole-genome sequencing (WGS), to achieve highly sensitive detection of CNVs in ctDNA (Katsman et al. 2022). The long reads uniquely allowed the simultaneous assessment of CNVs along with methylation features. Importantly, TGS revealed a significant link between CNV burden and recurrence risk. Combining methylation data further enhanced recurrence prediction.

#### Immune monitoring and recognition of rejection reactions

After liver transplantation, comprehensive immune monitoring becomes essential for ensuring graft survival and function. Clonal tracking of immune cells through T-cell receptor (TCR) sequencing allows for more precise identification of alloreactive T-cell clones in transplant recipients, supporting timely rejection interventions and personalized adjustments in immunosuppression to improve graft outcomes (Savage et al. 2020). Cieslak et al. showed that Nanopore provides accurate and cost-effective TCR clonality analysis, comparable to Illumina sequencing, with strong correlations in tumor clone detection (range R: 0.992–0.996; range r2: 0.984–0.991). Its rapid workflow makes it a promising tool for routine diagnostics (Cieslak et al. 2024).

Rejection reaction is a common and serious complication following liver transplantation, and early detection is vital to improving patient outcomes. TGS offers unique advantages in monitoring liver transplant rejection through its long-read capability and high sensitivity. By detecting subtle changes in donor-derived cell-free DNA (dd-cfDNA), TGS can identify rejection risks earlier than traditional liver function tests or imaging (Levitsky et al. 2022). This technology not only allows for dynamic monitoring of immune damage in the transplanted liver but also incorporates epigenetic analysis (such as DNA methylation) to reveal potential immune activation status, thereby helping identify high-risk patients before clinical symptoms emerge.

Moreover, liver transplant recipients are at high risk for opportunistic infections due to immunosuppressive therapy. Traditional diagnostic methods (e.g., culture, PCR, or short-read sequencing) may miss low-abundance pathogens or fail to resolve complex microbial communities. TGS technologies allow for rapid and accurate detection of opportunistic infections (e.g., CMV, EBV, and drug-resistant bacteria) (Deng et al. 2022; Chen and Xu 2023) while helping to distinguish infection from allograft rejection, which remains an ongoing diagnostic challenge in post-transplant care (Idossa and Simonetto 2017). This emerging monitoring technology not only increases the success rate of transplantation but also offers new hope for enhancing the long-term survival of liver cancer patients.

#### Analysis of drug resistance mechanisms

The TGS technology is also a powerful tool for studying drug resistance mechanisms in liver transplantation for hepatocellular carcinoma. This innovative approach allows comprehensive detection of genomic changes linked to resistance to targeted therapies such as sorafenib and Lenvatinib, including VEGFR2 and FGFR amplifications (Ladd et al. 2024). The long-read ability of this technology helps accurately identify complex genetic variations often involved in developing drug resistance, such as ASH1L rearrangements (Flynn et al. 2022) and TMPRSS2-ERG fusion genes (Poulsen et al. 2024), which frequently go undetected by traditional sequencing methods.

Another highly valuable application involves monitoring the real-time changes in drug-resistant mutations by analyzing ctDNA from patients after transplantation. For example, it can observe allele frequency shifts in PIK3CA mutations associated with mTOR inhibitor resistance, giving clinicians crucial molecular insights for timely treatment adjustments.

Furthermore, the technology’s unique ability to integrate multi-omics data, which includes genomic, epigenomic, and transcriptomic information, makes it a crucial part in developing personalized treatment plans for liver transplant patients with HCC. This comprehensive profiling offers unparalleled opportunities to understand and overcome therapeutic resistance in this difficult clinical setting.

#### Individualized adjuvant therapy

With the advancement of precision medicine, molecular typing based on TGS technology will become a key foundation for personalized treatment. In making treatment decisions for liver cancer patients, stratified intervention can be performed based on TGS results. For instance, patients in the high-risk group (such as those with high SV load or persistent positive ctDNA) might receive appropriate neoadjuvant therapy after liver transplantation (Singal et al. 2024). For patients with driver gene alterations (such as the presence of fusion genes or kinase activation), sequential targeted therapy may be considered, such as using MET or FGFR inhibitors. This approach can help achieve more precise therapeutic outcomes (Gujarathi et al. 2024). Furthermore, for patients with epigenetic dysregulation, combining demethylating drugs (like azacitidine) with local radiotherapy shows potential efficacy (Azad et al. 2017), offering diverse treatment options tailored to different molecular profiles.

## Challenges and improvement directions faced by TGS technology

### Sequencing error rate and data quality

One of the main challenges of TGS technology is its high error rate, which greatly affects the accuracy of the results. Vierstraete et al. found that when using the R9.4 MinION flow cell, the modal accuracy of the original read length falls between Q7 and Q12 quality scores, corresponding to an original single-base error rate of approximately 10% to 6% (Vierstraete and Braeckman 2022). This negatively impacts genome assembly, variant detection, and other downstream analyses. To improve sequencing data quality, researchers have developed various correction methods, aiming to use high-quality short-read data to correct errors in long-read data. For example, the NGSpeciesID tool improves the accuracy of consensus sequences by clustering and discarding reads with high error rates, thus reducing errors in downstream analyses (Sahlin et al. 2021). Another study shows that using the super-high-accuracy (SupHAC) mode of Guppy v5.0.7 significantly enhances the quality of the original read length, and the similarity between the consensus sequence and Sanger sequencing results is further improved (Buttler and Drown 2022). Additionally, reference-based correction methods combined with high-fidelity (HiFi) reads can greatly improve the accuracy of long-read data, thereby elevating overall data quality (Yu et al. 2024). Implementing these correction strategies effectively is essential for ensuring the reliability of TGS results.

### Data analysis and algorithm development

Handling long-read data generated by TGS presents substantial computational challenges. Due to the complexity of long-read data, traditional analysis tools often find it difficult to adapt to their unique characteristics. Wang et al. demonstrated that conventional short-read data analysis algorithms perform poorly with long-read data, leading to low efficiency and inaccurate results (Wang and Au 2020). Therefore, new computational algorithms and analysis strategies must be developed urgently to effectively address this issue. For example, the emerging framework Nano2NGS-Muta can convert long-read data into a short-read format. This conversion allows the use of existing short-read analysis pipelines for variant detection, significantly improving the accuracy and speed of the process (Lang et al. 2022). Additionally, with ongoing advancements in computing power and continuous algorithm optimization, future long-read data analysis will become more efficient, better supporting applications in fields such as genomics and transcriptomics.

To overcome the limitations of relying solely on either long-read or short-read data, researchers are exploring hybrid analysis strategies. These strategies combine the high accuracy of short-read data with the comprehensive sequence information of long-read data. For example, using long-read data for genome assembly and short-read data for error correction can significantly improve the accuracy and completeness of the final assembly (Chen et al. 2023). Additionally, hybrid analysis methods that merge these two data types can enhance the detection of complex structural variations, leading to a better understanding of genome structure. As algorithms and software tools continue to develop, future hybrid strategies will more effectively support genomic research.

### Cost and operational barriers

The rapid advancement of TGS technologies has been accompanied by decreasing costs of equipment and consumables, boosting their feasibility for broader research and clinical applications. ONT, for instance, has gained popularity due to its portability and affordability (Liu et al. 2024b). Increasing market competition has also driven the creation of more affordable sequencing options, making TGS more accessible to researchers and healthcare providers.

Concurrent improvements in workflow simplification and automation have greatly reduced operational complexity. Automated sample preparation and sequencing systems not only boost throughput but also reduce human errors (Vierstraete and Braeckman 2022). These advancements lower technical barriers, making it easier for non-specialists to adopt TGS. Standardized and modular protocols further enhance efficiency and reproducibility, providing more reliable data output for genomic studies and precision medicine.

### Clinical implementation barriers

Despite its transformative potential, integrating TGS into routine LT workflows faces significant clinical implementation barriers. First, turnaround time constraints present a major challenge: current TGS protocols require 24–48 h for library preparation and sequencing, while urgent clinical decisions often need results within 24 h. This time gap limits real-time use, although emerging rapid Nanopore kits show promise for bridging it. Additionally, interpretation challenges stem from the complexity of long-read data. About 40% of structural variations detected by TGS are novel or poorly understood variants with unknown clinical significance (Fujimoto et al. 2021), requiring specialized bioinformatics skills that most transplant centers currently lack. This uncertainty hampers clinical decision-making, especially for rare fusion transcripts or noncoding SVs. Finally, regulatory approvals are limited: currently, no TGS testing methods for liquid biopsy or transplant rejection monitoring of HCC have received approval from the US Food and Drug Administration (FDA) or certification under the European Medical Device Directive (CE-IVD). This confines their use to research settings. The lack of standardized analytical validation frameworks for long-read technologies also hinders laboratory-developed test (LDT) implementation, forcing institutions to develop costly internal validation protocols. Overcoming these barriers requires collaborative efforts to create streamlined TGS workflows, AI-assisted variant interpretation tools, and harmonized regulatory standards specifically designed for long-read diagnostics.

## The future development trend of third-generation sequencing technology

### Multidisciplinary integration promotes the advancement of precision medicine

The progress of TGS technology relies not only on developing the technology itself but also on the collaborative efforts of clinical, basic research, and computational science. By integrating multiple disciplines, a more comprehensive understanding of how liver cancer occurs can be achieved, providing a stronger theoretical foundation and data support for precision medicine (Athanasopoulou et al. 2022). For example, combining genomic and transcriptomic research helps uncover the molecular features of liver cancer cells, offering a solid basis for personalized treatment. Therefore, encouraging cooperation among different fields will be crucial for advancing the level of precision medicine.

With the application of TGS technology, researchers have also made significant progress in developing new treatment strategies. By conducting in-depth analysis of the genomes of liver cancer patients, potential targets for targeted therapy and immunotherapy can be identified, thereby offering patients more personalized treatment options (Medhi et al. 2025). Simultaneously, the development of new strategies also requires combining clinical trial data with findings from basic research to achieve more effective therapeutic results.

### AI-enhanced data analysis

Advanced machine learning and deep learning algorithms are transforming TGS by enhancing data interpretation efficiency and accuracy. Artificial intelligence (AI)-driven automation enables quick genomic analysis, biomarker detection, and clinical decision support while improving reproducibility (Wang et al. 2024b). These intelligent analytics also support clinical translation, helping physicians interpret genomic data for personalized treatment plans. In oncology and genetic disease diagnostics, comprehensive long-read sequencing combined with AI-driven insights will speed up progress in precision medicine (Guan and Quek 2025).

### Clinical transformation pathway

To accelerate the clinical adoption of TGS in LT for HCC, establishing multi-center cohorts for prospective validation is essential for thoroughly evaluating biomarkers such as structural variation signatures and methylation tags in prognosis and clinical decision-making (Moldogazieva et al. 2021). Systematic clinical trials must also address regulatory challenges, including the lack of FDA/CE-IVD-approved TGS assays, by developing standardized analytical validation frameworks that meet IVD standards. Additionally, ethical considerations require clear protocols for handling incidental germline findings (like cancer predisposition variants) during tumor genomic profiling, ensuring compliance with informed consent and data privacy guidelines. At the same time, clinical validation must overcome real-world barriers, such as interpreting new structural variants with unknown clinical significance and ensuring equitable access to TGS-based diagnostics. As these technologies become more widely used, establishing consistent laboratory procedures and clinical guidelines will be vital to ensure reproducibility, comparable results across institutions, and ultimately, the integration of TGS advancements into routine transplant care (Athanasopoulou et al. 2022).

## Conclusion

**Fig. 2:**
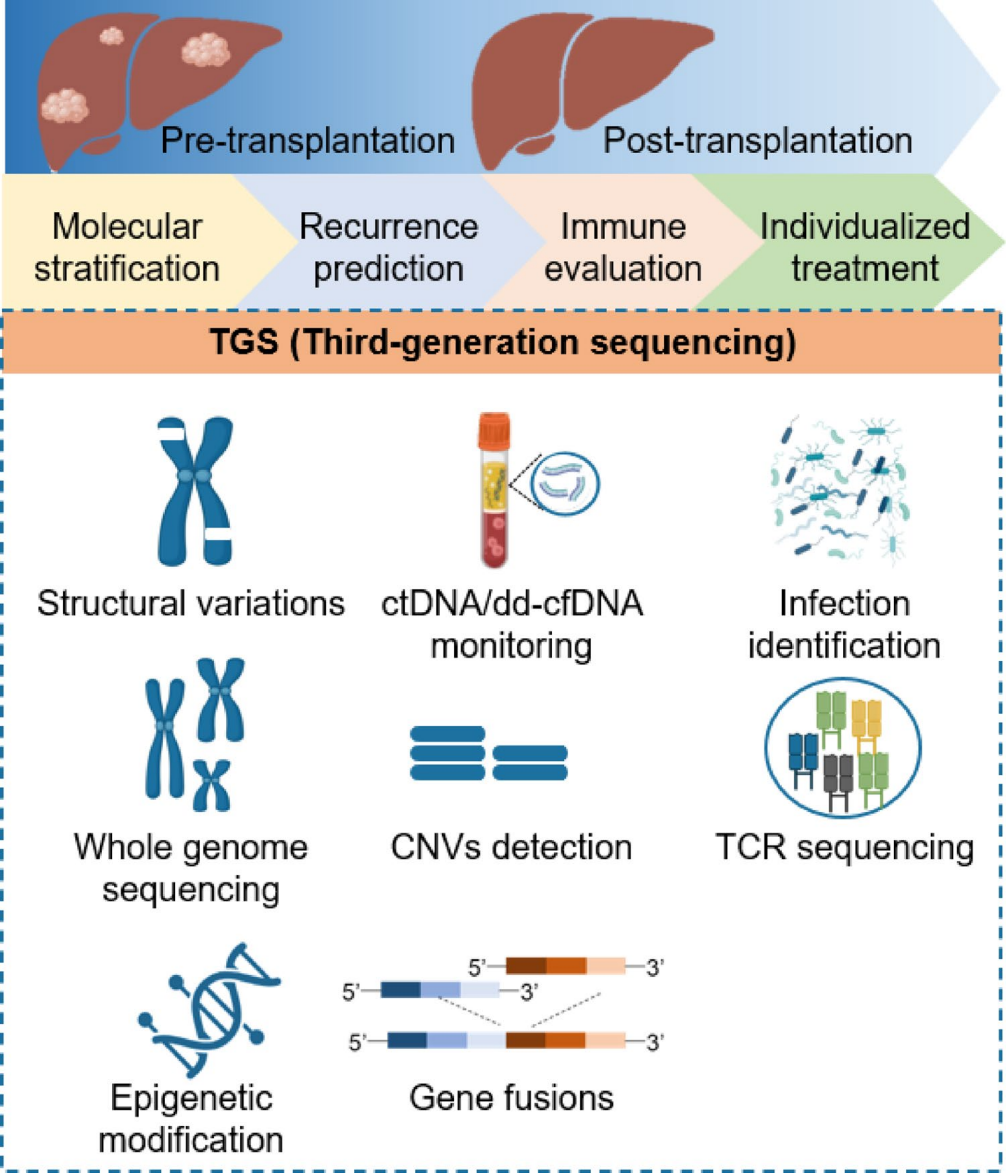
The application of TGS technology in liver transplantation for liver cancer. TGS technology shows promising clinical roles in HCC before and after liver transplantation, including molecular stratification, recurrence prediction, immune assessment, and personalized adjuvant therapy

This review systematically summarizes the potential of TGS in improving the diagnosis and management of HCC in liver transplant recipients (Fig. 2), as well as the associated biomarkers and clinical outcomes (Table 1). By enabling comprehensive structural variant profiling, epigenetic modulation analysis, and long-read liquid biopsy monitoring, this technology is transforming clinical decision-making. The integration of molecular stratification with traditional Milan criteria offers a new perspective on the selection criteria for liver transplant patients. This not only increases the chances of patients receiving liver transplants but also provides a more precise basis for clinical decision-making.

**Table 1:** TGS biomarkers for clinical decision-making in LT-HCC

TGS application	Key biomarkers	Clinical outcomes	References
Structural variation	SV burden, gene fusion (LINE1-MET, HBV-transposable element chimeras, ASH1L rearrangements, TMPRSS2-ERG)	Molecular stratification, discovery of drug-resistant mutations, adjuvant therapy	Fujimoto et al. (2021), Kiyose et al. (2022), Poulsen et al. (2024)
MVI detection	Methylation tags, ctDNA	Early recurrence risk prediction	Han et al. (2024), Afflerbach et al. (2024)
Liquid biopsy	ctDNA variants, CNV burden, methylation features, dd-cfDNA	Recurrence warning, treatment response, rejection prediction	Marcozzi et al. (2021), Katsman et al. (2022), Levitsky et al. (2022)
TCR sequencing	TCR rearrangement	Immune monitoring	Cieslak et al. (2021)
Pathogen identification	CMV, EBV, drug-resistant bacteria	Infection monitoring	Deng et al. (2022), Chen and Xu (2023)

In post-transplant management, using long-read ctDNA monitoring provides an innovative way to detect HCC recurrence early. This approach helps clinicians spot potential relapse risks before tumor recurrence happens, enabling timely intervention and greatly enhancing post-transplant survival rates. Additionally, immune monitoring and rejection assessment have become vital parts of post-transplant care. The move from traditional morphological evaluation to integrated molecular subtyping signifies a shift toward more precise and personalized practices in liver transplantation.

Although TGS has great potential to improve HCC management, challenges like cost, data processing, and clinical use still exist. The high cost of the technology limits its widespread adoption, and the complexity of analyzing data and applying it in clinical settings creates additional hurdles. Therefore, future research should aim to better integrate multi-omics data and translate findings into practical clinical strategies.

To enhance the clinical value of TGS, interdisciplinary collaboration is crucial. Close cooperation among clinicians, basic researchers, and bioinformaticians will improve the technology’s practical use. Additionally, as knowledge of HCC pathogenesis advances and new therapeutic targets are identified, precision treatment will become increasingly personalized, leading to better patient outcomes.

Looking ahead, TGS shows great promise in the field of HCC liver transplantation. As technology continues to improve and clinical research deepens, HCC treatment will shift from traditional methods to precision medicine, providing patients with better survival rates and quality of life. Ultimately, the rapid progress of this technology not only enhances HCC care but also reflects the broader movement towards precision medicine, marking a new chapter in healthcare.

## Data Availability

No datasets were generated or analysed during the current study.

## Abbreviations

TGS: Third-generation sequencing
HCC: Hepatocellular carcinoma
LT: Liver transplantation
UCSF: University of California, San Francisco
SMRT: Single-molecule real-time sequencing
ONT: Oxford Nanopore Technologies
ctDNA: Circulating tumor DNA
TCR: T-cell receptor
AI: Artificial intelligence
dd-cfDNA: Donor-derived cell-free DNA
CNVs: Copy number variations
NAHR: Non-allelic homologous recombination
ZMWs: Zero-mode waveguides
